# Expression Analysis of Oxalate Metabolic Pathway Genes Reveals Oxalate Regulation Patterns in Spinach

**DOI:** 10.3390/molecules23061286

**Published:** 2018-05-27

**Authors:** Xiaofeng Cai, Chenhui Ge, Chenxi Xu, Xiaoli Wang, Shui Wang, Quanhua Wang

**Affiliations:** Development and Collaborative Innovation Center of Plant Germplasm Resources, Shanghai Engineering Research Center of Plant Germplasm Resources, College of Life and Environment Science, Shanghai Normal University, Shanghai 200234, China; gechenhui@shnu.edu.cn (C.G.); chenxixu@shnu.edu.cn (C.X.); wxl2006by@163.com (X.W.); shuiwang@shnu.edu.cn (S.W.)

**Keywords:** spinach, oxalate, qRT-PCR, ammonium, nitrate

## Abstract

Spinach (*Spinacia oleracea* L.) is one of most important leafy vegetables because of its high nutritional value and high oxalate content, which can be toxic with negative effects on human nutrition. Ammonium and nitrate can effectively regulate oxalate accumulation, although the mechanisms underlying the oxalate biosynthesis and regulation are still undetermined in plants. In the present study, we identified 25 putative genes that are involved in the oxalate biosynthetic and degradation pathway, before analyzing the oxalate content and the expression levels of the corresponding proteins under normal growth conditions, with or without ammonium and nitrate treatments, using high and low oxalate-accumulated spinach genotypes. The two cultivars exhibited different profiles of total oxalate and soluble oxalate accumulation. The high oxalate concentrations in spinach were as a result of the high transcription levels of the genes that are involved in oxalate biosynthesis under normal growth conditions, such as *SoGLO2*, *SoGLO3*, three *SoOXACs*, *SoMLS*, *SoMDH1*, *SoMDH2*, and *SoMDH4*. The results revealed that the ammonium and nitrate were able to control the oxalate content in leaves, possibly because of the different transcription levels of the genes. The oxalate content is regulated by complex regulatory mechanisms and is varied in the different varieties of spinach. The results from this research may be used to assist the investigation of the mechanism of oxalate regulation and breeding for reduced oxalate content in spinach.

## 1. Introduction

Spinach (*Spinacia oleracea* L.) is widely cultivated as an economically important green leafy vegetable crop for consumption in both fresh and processed forms [1]. Spinach is produced in more than 50 countries, with production primarily occurring in China, USA, Japan, and Europe. The annual worldwide gross production of spinach was approximately 23 million tonnes in 2013, of which 91% was produced in China (FAOSTAT, http://faostat3.fao.org).

Spinach is considered to be one of the healthiest vegetables in the human diet because of its high concentration of nutrients and health-promoting compounds, such as beta carotene (provitamin A), lutein, folate, vitamin C, calcium, iron, phosphorous, and potassium [2,3]. However, spinach also contains a relatively large amount of oxalic acid compared to most crops [4,5,6,7,8], which affects both taste and human health. Oxalic acid plays an important biological function in plants, such as calcium regulation in plant cells [9], protection against herbivory [10], protection against pathogen defense response [11], tissue strength, light gathering, ion balance (e.g., Na and K) [12,13,14], and reflection [15,16]. Furthermore, Calcium oxalate crystals, an insoluble biomineral of oxalate in plants, may play a crucial role in the carbon cycle [17]. In addition, it has been reported that oxalate displays a positive role in the detoxifying of heavy metals, such as Al, by the formation of metal-oxalate complexes [12,18]. For human beings, oxalate is an anti-nutrient and an excessive consumption of oxalate-rich foods inhibits mineral absorption or creates calcium oxalate, which increases the risk of some diseases, such as kidney stones, in the digestive systems [19,20,21]. Thus, understanding the mechanism of oxalate accumulation, and aiming for the reduction of oxalate content in spinach has become a major concern, in terms of its potential health hazards to humans.

During the past decades, the genetic variation of oxalate concentrations in spinach has been extensively studied. Oxalate can be found in the form of soluble or insoluble oxalates in the leaves, which forms the total oxalates. The soluble oxalates can bind to Na^+^, K^+^, and NH_4_^+^ and the insoluble oxalates are only bound to Ca^2+^, Mg^2+^, and Fe^2+^ [22], the calcium oxalates have been observed to be located in the vacuole of the cell [15]. Insoluble oxalates are less likely to be absorbed in the digestive tract [19]. It has been demonstrated that there are significant differences in oxalate content. The spinach genotypes fall into a near normal distribution, which is in the range of 400–1700 mg/100 g on a fresh weight basis, or 5–15% on a dry weight basis [4,5,6,7,8], among them about 20–80% existed in insoluble form [22]. In general, the oxalates are mainly accumulated in leaves and least accumulated in the stems [19]. Furthermore, the oxalate concentrations in spinach are also related to the tissues, growth habits, amount of nutrients, light intensities, and growing season [5,7,23,24,25].

The nutrient nitrogen is one of the most important agronomic factors affecting the oxalate content in plants. Previous reports have indicated that the application of nitrogen of different forms, such as nitrate (NO_3_^−^) and ammonium (NH_4_^+^), could regulate the oxalate levels in plants [24,26,27]. The plants that had been treated with NO_3_^−^ were found have accumulated higher oxalate levels than those that had been exposed to NH_4_^+^, while NH_4_^+^ exposure significantly reduced the oxalate accumulation in plants by inhibiting the uptake of nitrate [24,26,27]. The NO_3_^−^ inhibited the enzymatic activity of oxalic acid oxidase and resulted in the accumulation of oxalic acid in the leaves and stems [28,29]. Furthermore, the different forms of nitrogen could influence the accumulation of glyoxylate, which was one of the precursors of oxalate, and appeared to affect the aggregation state of glycolate oxidase [28]. These findings will allow breeders to select spinach cultivars with low oxalate levels, or to reduce the oxalate levels in spinach during cultivation. However, the molecular mechanism of how NO_3_^−^ and NH_4_^+^ regulated the oxalate accumulation, with or without nitrogen, in spinach remains unknown.

**Figure 1:**
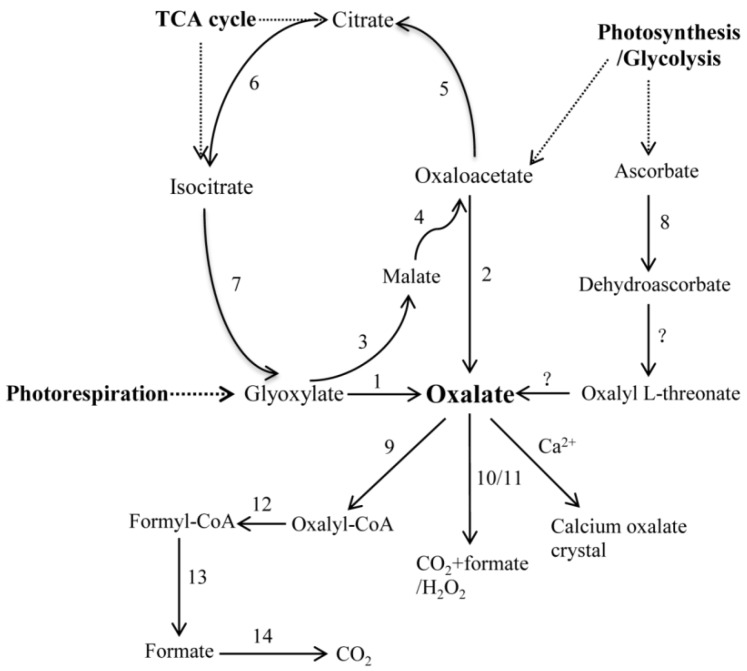
Schematic representation of putative biosynthetic and the degradation pathway of the oxalate (Cai et al., 2015). The enzyme names are as follows: (1) Glycolate oxidase (GLO); (2) Oxaloacetate acetylhydrolase (OXAC); (3) Malate synthase (MLS); (4) Malate dehydrogenase (MDH); (5) Citrate synthase (CTS); (6) Aconitase (ACO); (7) Isocitrate lyase (ICL); (8) Ascorbate peroxidase/Ascorbate oxidase (APX/AO); (9) Oxalyl-CoA synthetase (AAE3); (10) Oxalate decarboxylase (OXDC); (11) Oxalate oxidase (OXO); (12) Oxalyl-CoA decarboxylase (OXDE); (13) Formyl-CoA hydrolase (FXH); and (14) Formate dehydrogenase (FXDE).

Until now, the oxalate biosynthetic and degradation pathway has not been well-documented, and there are only a few genes that are responsible for the oxalate metabolism that have been functionally identified in plants. However, it has been proposed that oxalate is synthesized via three precursors, which are glyoxylate/glycolate, ascorbate, and oxaloacetate (Figure 1) [30], although none of them have been conclusively proven to be involved in the biosynthesis of oxalate [31,32,33]. The oxidation of glycolate/glyoxylate has been seen as being part of a biosynthetic pathway for oxalate, during photorespiration and the glyoxylate cycle in plants, which is catalyzed through glycolate oxidase (GLO) [31,34]. The oxidative degradation of oxaloacetate was hypothesized to be the third source for oxalate in plants, which was presumably catalyzed by oxaloacetate acetylhydrolase (OXAC) [35]. The participating genes included malate synthase (MLS), malate dehydrogenase (MDH), citrate synthase (CTS), aconitase (ACO), and isocitrate lyase (ICL). The biochemical measurements, which were obtained using radiolabeled precursors, supported a C2/C3 cleavage of ascorbic acid as a major pathway of oxalate production [36,37,38], however the genes that are involved in this pathway have not been identified. Thus, the mechanisms underlying oxalate biosynthesis and regulation are still undetermined in plants.

The degradation of oxalate occurs through oxidation, decarboxylation, and acetylation (Figure 1) [30]. Oxalate oxidase (OXO), which is one of the most important oxalate-degrading enzymes, breaks down oxalate to carbon dioxide and hydrogen peroxide. Oxalate decarboxylase (OXDC) catalyzes oxalate to form carbon dioxide and formic acid [39,40]. Moreover, oxalic acid can also generate oxalyl-CoA by the catalysis of oxalyl-CoA synthetase (AAE3), which can be finally degraded to carbon dioxide through three enzymatic reactions. These three reactions are catalyzed by oxalyl-CoA decarboxylase (OXDE), formyl-CoA hydrolase (FXH), and formyl-CoA dehydrogenase (FXDE) [41]. Oxalic acid can also be combined with calcium to form calcium oxalate crystals in plants.

Recently, the spinach genome and transcriptome was sequenced and assembled de novo, which has provided an important tool for identifying and classifying the genes that are involved in the oxalate metabolism pathway [42,43]. In this present study, we investigated the accumulation of oxalate in different parts of two spinach varieties and performed a comparative transcription analysis of the putative oxalate-related genes between these two spinach varieties under normal growth conditions, with or without ammonium and nitrate treatments. The experimental results may facilitate a better understanding of the oxalate biosynthesis and metabolism, which can ultimately help in developing breeding strategies so as to reduce oxalate levels in spinach.

## 2. Results

### 2.1. Oxalate Accumulation under Normal Growth Conditions in Spinach

**Figure 2:**
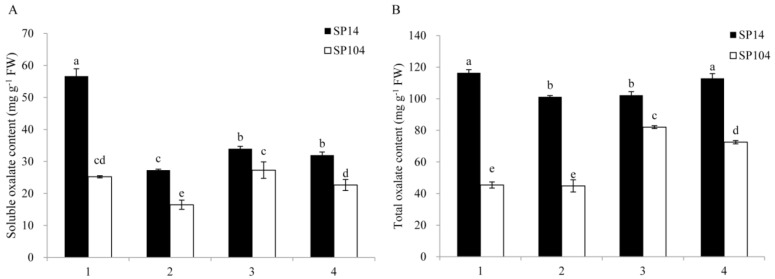
Contents of soluble oxalate (**A**) and total oxalate (**B**) in four parts of spinach. The four parts were (1) mature leaf laminas; (2) mature leaf petioles; (3) young leaf laminas; and (4) young leaf petioles. Error bars represent standard deviation among three independent replicates. Data are means of three replicates ±SD. Different letters (a, b, c, or d) indicate significant differences at *P* < 0.05.

The soluble and total oxalate contents were determined in four spinach parts (mature leaf lamina, mature leaf petiole, young leaf lamina, and young leaf petiole) of two spinach varieties (SP14 and SP104) under normal growth conditions (Figure 2). The soluble and total oxalate contents in all of the detected spinach parts were significantly higher in the high oxalate-accumulated spinach cultivar SP14 compared with the low oxalate-accumulated spinach cultivar SP104. The soluble oxalate contents in the mature leaf lamina and mature leaf petiole of SP14 were 56.7 mg/g fresh weight (FW) and 27.3 mg/g FW, which was 2.25- and 1.66-fold higher than that in SP104, respectively. The soluble oxalate contents in the young leaf lamina and leaf petiole of SP14 were 34.0 mg/g FW and 32.0 mg/g FW, which was 1.25- and 1.41-fold higher than that in SP104, respectively. Furthermore, the soluble oxalate contents in the leaf laminas were remarkably higher than that in petioles, with slight differences between the young leaf laminas and petiole, while the soluble oxalate was mainly accumulated in the leaf laminas (Figure 2A).

The total oxalate contents in the mature leaf lamina and mature leaf petiole of SP14 were 116.4 mg/g FW and 101.2 mg/g FW, which was 2.56- and 2.26-fold higher than that in SP104, respectively. The total oxalate contents in the young leaf lamina and leaf petiole of SP14 were 102.2 mg/g FW and 112.9 mg/g FW, which was 1.25- and 1.56-fold higher than that in SP104, respectively. In addition, the total oxalate contents in all of the detected parts of SP14 were slightly different from each other, although the total oxalate contents in the young leaf laminas and petioles of SP104 were remarkably higher than those in the leaf laminas and leaf petioles (Figure 2B).

### 2.2. Oxalate Accumulation under NO_3_^−^ and NH_4_^+^ Growth Conditions in Spinach

**Figure 3:**
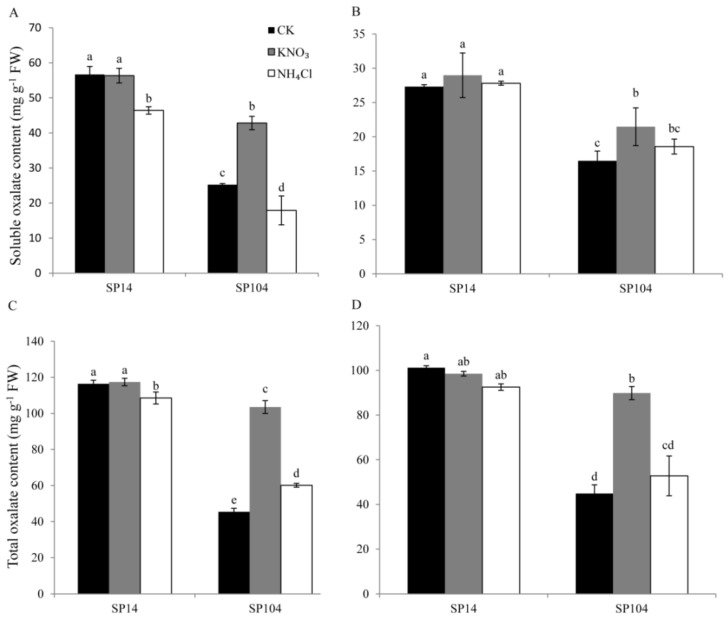
Contents of soluble oxalate (**A**,**B**) and total oxalate (**C**,**D**) in the leaf laminas (**A**,**C**) and leaf petioles (**B**,**D**) of two spinach cultivars in response to NH_4_^+^ and NO_3_^−^ treatments. Error bars represent standard deviation among three independent replicates. The data are the means of three replicates ±SD. Different letters (a, b, c, or d) indicate significant differences at *P* < 0.05.

Under the NO_3_^−^ and NH_4_^+^ growth conditions, the soluble and total oxalate contents were determined in the mature leaf lamina and mature leaf petiole of two spinach varieties (Figure 3). In the high oxalate-accumulated spinach cultivar SP14, the soluble and total oxalate contents in the leaf laminas were significantly reduced under the NH_4_^+^ growth conditions when they were compared to the control (Figure 3A,C), which was 0.82- and 0.91-fold lower than the control, respectively. There was a smaller effect found on the soluble and total oxalate contents under the NO_3_^−^ growth conditions in the leaves and under the NO_3_^−^ and NH_4_^+^ growth conditions in the leaf petioles (Figure 3B,D). In the low oxalate-accumulated spinach cultivar SP104, remarkable increases in the soluble and total oxalate contents were detected in the leaf laminas and leaf petioles under the NO_3_^−^ treatment, when compared to the control (Figure 3A,C). In contrast, the reduced soluble oxalate contents were found after the NH_4_^+^ treatment in the leaf laminas, while no significant changes were detected under the NO_3_^−^ and NH_4_^+^ growth conditions in the leaf petioles (Figure 3B,D).

### 2.3. Identification of Putative Oxalate Biosynthesis and Metabolism Genes

All of the putative genes that were involved in the oxalate biosynthesis and metabolism pathway were identified, except for formyl-CoA hydrolase, while 25 putative genes were classified and identified in spinach, based on the spinach genomic and transcriptomic database. The names and the unigene numbers of the identified genes, the length of open reading frame (ORF), amino acids, genomic location, and number of introns are shown in Table 1.

There were five, three, four, and two putative *SoGLO*, *SoOXAC*, *SoMDH*, and *SoCTS* genes that were involved in the oxalate biosynthesis in spinach, respectively. There were three and two putative *SoOXDC* and *SoOXO* genes that were involved in the oxalate degradation in spinach, respectively. The remaining putative genes had one copy. Of the 25 oxalate-related genes, 19 were mapped to the 5 chromosomes of spinach, with 2, 3, 2, 8, and 4 oxalate-related genes located on chromosomes 1–5 of the spinach genome, respectively (Table 1). The remaining six genes were anchored on six different scaffolds, which were not yet been mapped onto a chromosome.

### 2.4. Expression of Putative Genes Involved in Oxalate Biosynthesis in Spinach

**Figure 4:**
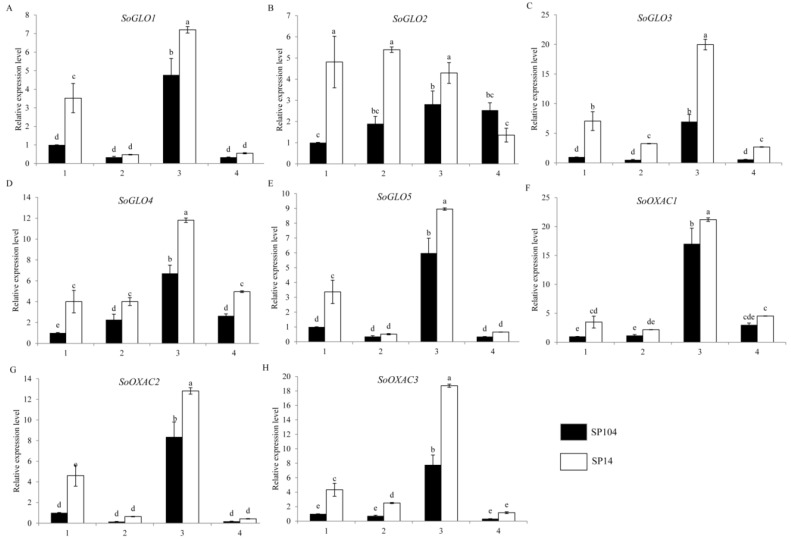
Expression profiles of putative genes involved in oxalate biosynthesis pathways in spinach. The four parts were as follows: (1) mature leaf laminas; (2) mature leaf petioles; (3) young leaf laminas; and (4) young leaf petioles. Error bars represent standard deviation among three real-time quantitative PCR reaction replicates. Data are means of three replicates ±SD. Different lowercase letters indicate significant differences at *P* < 0.05.

To investigate the molecular mechanism of oxalate accumulation, the expression profiles of the putative oxalate biosynthetic genes in the four parts of the two spinach varieties were examined. Glycolate oxidase (GLO) was one of the key genes, which catalyzed the synthesis of the oxalate in the plant. The expression patterns of five putative glycolate oxidase (GLO) genes showed that the *SoGLO2*, *SoGLO3*, and *SoGLO4* had higher levels of expression in four parts of SP14 compared to SP104, with an increase that was at least 3-fold in the mature leaf laminas (Figure 4B–D) and showed similar trends with the oxalate content (Figure 2). The *SoGLO1* and *SoGLO5* had higher levels of expression in the leaf laminas of SP14 compared to SP104 (Figure 4A,E). In addition, the expression of *SoGLO1*, *SoGLO3*, *SoGLO4*, and *SoGLO5* exhibited extremely high levels of expression in the young leaf laminas (Figure 4A,C–E), while the gene of *SoGLO2* had a high level of expression in the four parts of SP14 (Figure 4B). Furthermore, the highest expression was detected in the mature leaf laminas and petioles of *SoGLO3* in SP14, which were 7.0- and 6.2-fold higher compared to SP104, respectively (Figure 4C).

Oxaloacetate acetylhydrolase (OXAC) was another key gene, which degraded oxaloacetate to synthetic oxalate. There were three homologous putative genes of *SoOXAC* in spinach, while the expression patterns of *SoOXAC1*, *SoOXAC2*, and *SoOXAC3* were analyzed. The expression level of the *SoOXAC1* and *SoOXAC2* genes were higher in the mature and young leaf laminas of SP14 compared to SP104, in which the expression level of *SoOXACs* was 3- to 4-fold higher. There were also slight changes in the mature and young leaf petioles (Figure 4F,G), while the *SoOXAC3* gene showed a significant difference in the mature leaf laminas, mature leaf petioles, and young leaf laminas of SP14 compared to SP104 (Figure 4H).

The intermediate metabolites of the glycolate cycle were also used in the oxalate synthesis in plants, and thus, we quantified the transcription of the genes that were involved in the glycolate cycle. The expression patterns of *SoMLS* showed the same variation trends in *SoGLO2*, *SoGLO3*, and *SoGLO4* (Supplementary Figure S1H). The transcription levels of *SoMDH2*, *SoMDH4*, and *SoCTS2* in the mature and young leaf laminas of SP14 were higher compared to SP104 (Supplementary Figure S1B,D,F), in which these two genes showed 3- to 4-fold changes. In contrast, *SoMDH1* and *SoACO* exhibited the highest amount of mRNA in the mature leaf laminas and mature leaf petioles (Supplementary Figure S1C,G). The remaining genes were slightly changed in the SP14, compared to SP104 (Supplementary Figure S1A).

### 2.5. Expression of Putative Oxalate Degradation Genes in Spinach

**Figure 5:**
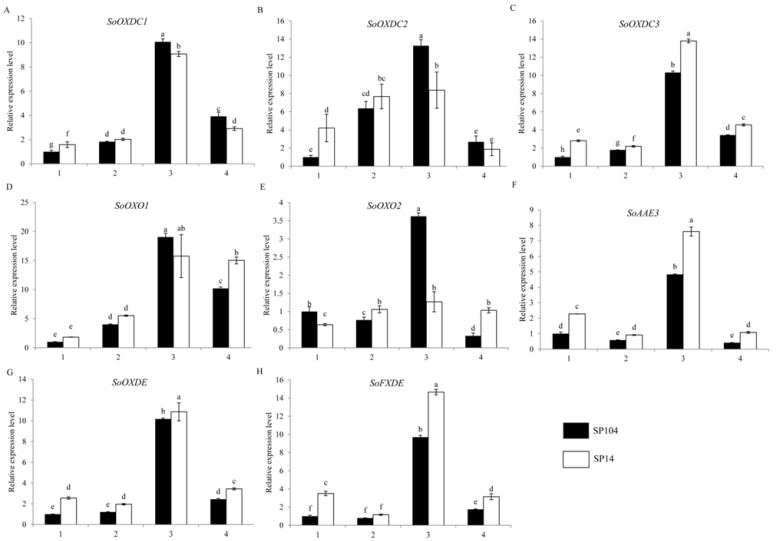
Expression profiles of putative genes involved in oxalate degradation pathways in spinach. The four parts were as follows: (1) mature leaf laminas; (2) mature leaf petioles; (3) young leaf laminas; (4), young leaf petioles. Error bars represent standard deviation among three real-time quantitative PCR reaction replicates. Data are means of three replicates ±SD. Different lowercase letters indicate significant differences at *P* < 0.05.

The oxalate oxidase, oxalate decarboxylase, and oxalyl-CoA were found to catalyze the degradation of oxalate, so the levels of transcription of the putative *SoOXO*, *SoOXDC*, and *SoAAE3* were analyzed (Figure 5). The results suggested that the expression levels of *SoOXDC1* and *SoOXDC*2 were lower in the young leaf laminas of SP14, while these had higher levels of expression in the mature leaf laminas of SP14 (Figure 5A,B). Furthermore, *SoOXDC*3 was highly expressed in the four parts of SP14 (Figure 5C); *SoOXO*2 had a low level of expression in the leaf laminas of SP14, and a high level of expression in the petioles of SP14 (Figure 5E); and *SoOXO1* was not significantly changed in SP14 and SP104 (Figure 5D). The expression of the *SoAAE3* gene was higher in the four detected parts of SP14, compared with SP104, which showed a 1.5-fold change (Figure 5F). The following acetylated genes exhibited similar expression patterns with the *SoAAE3* gene (Figure 5G,H).

### 2.6. Expression of Putative Oxalate Related Genes under NO_3_^−^ and NH_4_^+^ Treatments

**Figure 6:**
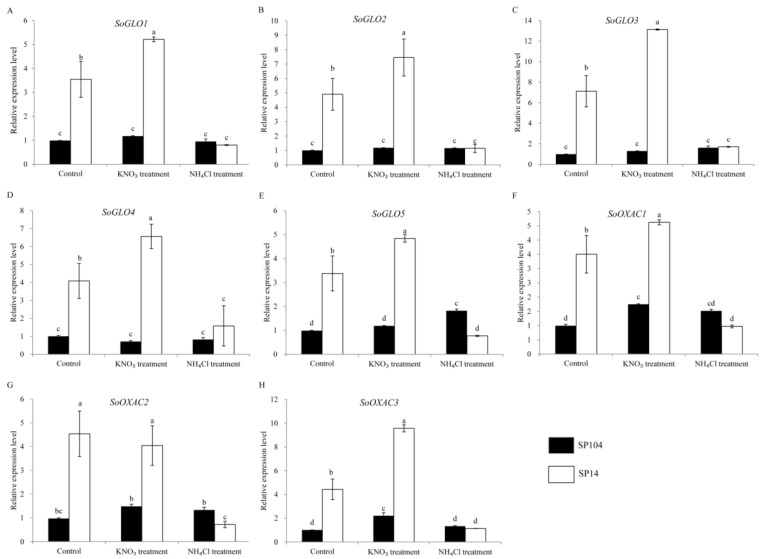
Expression profiles of putative genes involved in the oxalate biosynthesis pathways response to NH_4_^+^ and NO_3_^−^ treatments in the leaf laminas of spinach. Error bars represent standard deviation among three real-time quantitative PCR reaction replicates. Data are means of three replicates ±SD. Different lowercase letters indicate significant differences at *P* < 0.05.

**Figure 7:**
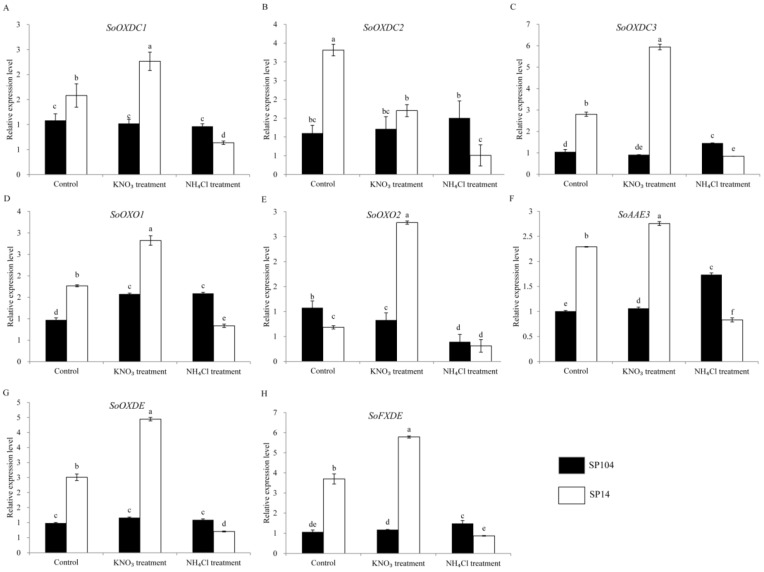
Expression profiles of the putative genes involved in the oxalate degradation pathways response to NH_4_^+^ and NO_3_^−^ treatments in the leaf laminas of spinach. Error bars represent standard deviation among three real-time quantitative PCR reaction replicates. Data are means of three replicates ±SD. Different lowercase letters indicate significant differences at *P* < 0.05.

To investigate the molecular mechanism of oxalate accumulation under NO_3_^−^ and NH_4_^+^ treatments, the expression profiles of the putative oxalate related genes in the leaf laminas of two spinach cultivars were examined. In the leaf laminas of the high oxalate accumulation cultivar SP14, all of the oxalate-related genes exhibited the remarkably upregulated expression under NO_3_^−^ treatments, with 1- to 2-fold changes compared with the controls. In contrast, these genes displayed significantly downregulated expressions in response to the NH_4_^+^ treatments, with a reduction of 70–80% compared with the controls (Figure 6 and Figure 7 and Supplementary Figure S2). The expression patterns are not consistent with the amount of oxalate production in the corresponding parts (Figure 3).

In the leaf laminas of low oxalate accumulation cultivar SP104, all of the putative *SoGLOs* genes exhibited slightly changed expression levels under the NO_3_^−^ and NH_4_^+^ treatments (Figure 6A–E). In contrast, the key genes of *SoOXAC1* and *SoOXAC3* that were involved in the degradation of oxaloacetate to oxalate displayed a significantly upregulated expression in response to the NO_3_^−^ treatments, with 1.5- to 2.1-fold changes compared to controls. These showed no differences after the NH_4_^+^ treatments (Figure 6F–H). Furthermore, the transcription level of the genes that were involved in the glycolate cycle, such as *SoCTS2*, *SoMDH1*, *SoMDH2*, *SoMDH3*, and *SoACO* were, significantly upregulated in response to the NO_3_^−^ treatments, while there was no difference after the NH_4_^+^ treatments. The remaining genes were slightly changed under the NO_3_^−^ and NH_4_^+^ treatments (Supplementary Figure S2). The transcription level analysis of the oxalate degradation pathway genes suggested that the expression levels of *SoAAE3*, *SoFXDE*, *SoOXO1*, and *SoOXDC*3 were downregulated under the NH_4_^+^ treatments, while a lower expression level or no significant changes were found under the NO_3_^−^ treatments (Figure 7). In addition, the expression of the oxalate degradation pathway genes was not significantly raised in response to the NO_3_^−^ treatments (Figure 7).

## 3. Discussion

Oxalate is widely distributed in the plant kingdom, and many plant species have accumulated a large amount of oxalate, particularly in spinach, which accounts for 5–15% of a dry weight basis. It had been reported that oxalate might have played various functional roles in plants, including calcium regulation, ion balance (e.g., Na and K), and heavy metal detoxification [12,13,14]. Despite the positive functional roles of oxalate in plants, excess oxalate consumption in food crops was a concern as it could have had negative consequences on human health [19,20]. Consequently, it was significantly important for both the scientific and applied aspects in order to understand the oxalate metabolic and regulatory mechanisms in plants.

Biochemical and physiological approaches were used in previous studies to achieve great progress in investigating the genetic variation of oxalate concentrations in spinach. Several pathways for oxalate production were proposed, including glycolate/glyoxylate oxidation, cleavage of ascorbate, and hydrolysis of oxaloacetate (Figure 1) [30]. Nevertheless, there was still a lack of knowledge regarding the molecular biological regulation of oxalate accumulation in spinach. In the present work, we identified 25 putative genes (Table 1) from oxalate biosynthetic and degradative routes and studied their expression levels in two spinach cultivars (high-oxalate spinach and low-oxalate spinach) under normal growth conditions, with and without ammonium and nitrate (Figure 2). The results indicated that there was a strong and stable genetic basis for the regulation of the oxalate accumulation in spinach.

Glycolate/glyoxylate oxidation had long since been proposed as an important pathway for oxalate biosynthesis in plants [31,34], in which glycolate oxidase (GLO) was the key gene for the catalysis of glyoxylate oxidation to oxalate. There were five copies of *SoGLO* genes in spinach genome (Table 1), and the transcription level analysis suggested that the enhanced expression of the *SoGLO* genes were correlated with higher oxalate concentrations in the SP14 variety. In particular, the *SoGLO2*, *SoGLO3*, and *SoGLO4* genes had higher levels of expression and were strongly correlated with the oxalate levels in the SP14 variety (Figure 2 and Figure 4B–D). It was noticed previously that glycolate was able to effectively increase the oxalate accumulation by altering the feeding treatments of the leaves [34]. Therefore, these three *SoGLO* genes seemed to be represented as good candidates for the regulation of the oxalate concentrations in spinach. Furthermore, high expression levels of *SoMLS*, *SoMDH1*, *SoMDH2*, and *SoMDH4* in the mature or young leaf laminas were also detected (Supplementary Figure S1C,D,F,H), which indicated that these transcripts might have also played a role in regulating the oxalate accumulation.

An oxaloacetate breakdown was reported as the source of oxalate in plants [35], which was catalyzed by oxaloacetate acetylhydrolase (OXAC), and appeared to be an especially important route in fungi [44]. In this present study, three homologous genes of *SoOXAC* were found in spinach, and the differences in the expressions of *SoOXAC* genes could have also helped to explain the differences in the oxalate concentrations between the cultivars, as the expression levels were three or four times higher in the SP14 compared with the low-oxalate cultivar SP104 (Figure 4). Overall, these results indicated that the high oxalate concentrations in SP14 could have been attributed to the high transcription levels of the genes that were involved in oxalate biosynthesis.

The oxalate concentration might have been determined by both its biosynthesis and its degradation in spinach. Although *SoOXDC1*, *SoOXDC2*, and *SoOXO2* had low levels of expression in the young leaf laminas of SP14 (Figure 5A,B,E), these had high expression levels in the mature leaf laminas of SP14 (Figure 6). That might have been because of the highest levels of oxalate in the mature leaf laminas of SP14 (Figure 2). Oxalate could be toxic to plants and could induce programmed cell death in plant organs [45,46]. Consequently, the plant organs had to maintain a high expression level for the genes from the oxalate degradation pathway, in order maintain the steady state, so as to reduce the harm from the excess oxalate in the mature leaf laminas of SP14. The overexpression of the *OXDC*, *OXO*, or *AAE3* genes could remarkably reduce the oxalate content in plants [41,47,48]. Therefore, the oxalate degradation steps, especially the *SoOXDC*, *SoOXO*, or *SoAAE3* genes, appeared to be important factors in the regulation of oxalate content in spinach. Furthermore, a large amount of oxalate (about 60%) was found to be stored by an insoluble form, such as calcium oxalate, and the insoluble oxalates in the mature and immature leaf of SP14 were 2.78- and 1.43-fold higher than that in SP104, respectively (Figure 2 and Figure 3). The calcium oxalate not only played a very important role in changing the concentration of calcium but also in regulating the oxalate levels [12]. This finding suggested that calcium oxalate crystal formation could have been an efficient and economical way to sequester excess oxalate.

Many studies were conducted in recent years with the aim of reducing oxalate accumulation in plants, which have indicated that the nitrogen nutrient was one of the most important agronomic factors for effectively regulating the oxalate levels in plants. The plants that were treated with NO_3_^−^ accumulated higher oxalate levels than those that were exposed to NH_4_^+^, while the NH_4_^+^ exposure significantly reduced the oxalate accumulation in the plants by inhibiting the uptake of nitrate [24,26,27]. In this study, we found that the total, insoluble, and soluble oxalate contents in the leaves and leaf petioles of SP104 were significantly increased after NO_3_^−^ treatments, which showed a slight variation in the oxalate contents in the leaf laminas and leaf petioles of SP14 (Figure 3). Similar results were previously reported [24,26,27]. On the other hand, the NH_4_^+^ treatments did reduce the soluble oxalate contents, but they increased the insoluble oxalate in the leaf laminas of two spinach cultivars and the total oxalate contents in the leaf laminas of SP14 (Figure 3), while a smaller effect was found in other parts (Figure 3). Several reports showed that the oxalate accumulation in the leaves was decreased in the NH_4_^+^ solution or in the presence of a low NO_3_^−^/NH_4_^+^ ratio [23,49]. However, the connection between nitrogen, such as through NO_3_^−^ and NH_4_^+^ treatments, to the related genes expression and oxalate accumulation remained unknown in spinach.

To explore the possible mechanism that was involved in oxalate accumulation in response to ammonium and nitrate, the transcription levels of 25 proposed oxalate related genes were analyzed. The results suggested that NH_4_^+^ and NO_3_^−^ possessed opposite functions in oxalate accumulation and genes expression in spinach (Figure 3, Figure 6 and Figure 7). NH_4_^+^ induced the downregulation of oxalate metabolism genes, which was consistent with the lower oxalate content in the leaf laminas of SP14. Significantly higher expression levels were detected under NO_3_^−^ treatments (Figure 6 and Figure 7), although there was a smaller effect on the soluble and total oxalate contents in the leaf laminas of SP14 under the NO_3_^−^ growth conditions in the leaf laminas (Figure 3). This might have been because of the limitation of the reaction substrates or because of the high expression level of the genes from the oxalate degradation pathway, which resulted in the spinach maintaining a new steady state so as to reduce the harm of excess oxalate in the mature leaf laminas of SP14.

In contrast, in the low-oxalate spinach cultivar SP104, the increased oxalate contents were correlated with higher expression levels of *SoOXAC1* and *SoOXAC3*, and the genes that were involved in glycolate cycle under NO_3_^−^ treatments (Figure 6 and Figure 7), while there were lower expression levels of *SoAAE3*, *SoFXDE*, *SoOXO1*, and *SoOXDC3* under NH_4_^+^ treatments (Supplementary Figure S2). The results suggested that the oxaloacetate pathway could have been the major pathway response to NO_3_^−^ treatments and that acetylation was the major oxalate degradation pathway. Overall, the above results indicated that the oxalate contents were regulated by complex regulatory mechanisms and that they varied in the different varieties of spinach. However, further functional analysis, enzymatic activity assay, and identification of the promoter *cis*-acting elements that were localized upstream of the candidate genes, was necessary. Furthermore, more candidate genes or a locus and molecular marker would have been identified using quantitative trait loci (QTL) and association mapping.

## 4. Materials and Methods

### 4.1. Materials and Treatments

The seeds of the two spinach varieties, SP14 and SP104, were provided by Laizhou Seed Company (Shandong, China) and Jiuquan Suzhou Seed Company (Gansu, China), respectively. The seeds of the spinach were surface-sterilized using 1% (*w*/*v*) sodium hypochlorite for 10 min, washed three times, soaked in deionized water for 12 h, and germinated for 12 h at 4 °C. The uniform seeds were selected and sowed in perlite and vermiculite (1:1) in a green house with a 12 h photoperiod (300–400 μE m^−1^ s^−1^), day/night temperature regime of 25/15 °C and 75%/80% relative humidity. After about two weeks, after which the seedlings were transferred to a 25% full-strength Hoagland solution under standard green-house conditions, with a 10 h light (27 °C) and 14 h dark (15 °C) cycle. He nitrogen was supplied as 200 μM NO_3_^−^ or NH_4_^+^, with six plants per 1 L tank. The pH values of the solutions were adjusted to 6.0 and were maintained at this pH by the daily addition of 0.1 M HCl or NaOH. The nutrient solution was aerated continuously and completely renewed every two days. All of the experiments were conducted in three replications. The fresh, young, and healthy full expanding leaves were randomly collected from six seedlings of 50 day old plants, and were immediately frozen in liquid nitrogen and stored at −80 °C until use.

### 4.2. Determination of Oxalate Levels

A 0.2-g leaf sample was homogenized in 4 mL of deionized water and transferred to a 10-mL centrifugal tube for soluble oxalate analysis. The homogenate was heated at 80 °C (water bath) for 30 min with intermittent shaking. A total of 5 mL of deionized water was added when the homogenate had cooled, and the solution was kept overnight. About 1 mL of the solution was withdrawn and centrifuged at 12,000× *g* for 10 min. A total of 1 mL of the supernatant was passed through a filter (0.22 µm) before a high-performance liquid chromatography (HPLC) analysis. These conditions were also tested for the total oxalate, although the extraction was carried out by homogenization in 4 mL of 0.5 M HCl. The HPLC analysis was conducted according to Lin et al. [26]. The standard curves were prepared and used to quantitate the soluble and total oxalic acid contents. The oxalate contents were calculated and converted to mg/100 g fresh weight (FW) of the samples. The measurements of each sample were based on three replicates and the mean values were used.

### 4.3. Identification of Putative Oxalate Biosynthesis and Metabolism Genes

All of the sequences of the genes (Figure 1 and Supplementary Table S1) that were involved in the oxalate metabolism were downloaded from the NCBI database (https://www.ncbi.nlm.nih.gov/). The spinach genome and transcriptome sequences (http://www.spinachbase.org/cgi-bin/spinach/index.cgi) were used to identify the putative oxalate biosynthesis and metabolism genes in spinach, using BLASTN and BLASTP, with a cut off E-value ≤ 1 × 10^−10^ and a coverage ≥ 0.75. The blast results were confirmed by the best hits and the gene annotations. Only the non-redundant genes were identified and their uniqueness was manually verified by removing the redundant sequences from the databases and different transcripts of the same gene.

### 4.4. RNA Extraction and Real-Time qRT-PCR Analysis

The total RNA was extracted with TRIzol reagent (Invitrogen, USA) and was treated with RNase-free DNase I (Invitrogen, Gaithersburg, MD, USA). The first-strand cDNA was synthesized from 3 mg of the total RNA from each sample, using a high capacity cDNA reverse transcription kit (TOYOBO, Japan), according to the supplier's protocols. After this, the quantitative RT-PCR (qRT-PCR) was conducted, as described previously [50]. The spinach *So18s* gene was used as the internal control, and three independent replicates were performed. The real-time PCR data were analyzed using the 2^−∆∆T^ method. A total of 25 gene specific primer pairs for the oxalate relative genes were designed using Primer Premier 5, which were synthesized from Sangon Biotech (Shanghai) Co., Ltd. The primers that were used are listed in Supplementary Table S2.

### 4.5. Statistical Analysis

The statistical analysis was performed using Excel and SAS software. The significant differences were calculated using the Student’s *t*- or Levene’s F-test at a 95% confidence limit.

## 5. Conclusions

We identified 25 putative genes that were involved in the oxalate biosynthetic and degradation pathway, in addition to analyzing the oxalate content and the expression levels of the corresponding proteins under normal growth conditions, with or without ammonium and nitrate treatments. The two cultivars exhibited different profiles of total oxalate and soluble oxalate accumulation. The high oxalate concentrations in spinach were because of the high transcription levels of the genes that were involved in oxalate biosynthesis, under normal growth conditions, such as *SoGLO2*, *SoGLO3*, three *SoOXACs*, *SoMLS*, *SoMDH1*, *SoMDH2*, and *SoMDH4*. The ammonium and nitrate could control the content of oxalate in the leaves, possibly because of the different transcription levels of genes. The results from this research may be used to assist in the investigation of the mechanism of oxalate regulation, which will help in breeding for a reduced oxalate content in spinach.

## Figures and Tables

**Table 1:** Putative oxalate related genes and the information of each gene.

Num	Gene Name	Unigene Number	Chomosome/Scaffold and Location			Strand	CDS (bp)	Size (aa)	Introns
1	SoGLO1	Spo19861	chr4	118492560	118496915	Rev	1053	350	10
2	SoGLO2	Spo21903	chr5	35418377	35422331	For	1107	368	10
3	SoGLO3	Spo10076	chr1	45522012	45524813	Rev	1095	364	9
4	SoGLO4	Spo21282	SpoScf_01013	111890	113703	For	756	251	7
5	SoGLO5	Spo20781	chr4	118492935	118496947	Rev	720	239	7
6	SoOXAC1	Spo00571	SpoScf_01500	31009	43616	For	1875	624	9
7	SoOXAC2	Spo21624	chr2	55761708	55765402	Rev	909	302	4
8	SoOXAC3	Spo21589	chr2	55798280	55800798	For	915	304	4
9	SoMLS	Spo16696	chr5	17999221	18002655	For	1713	570	3
10	SoMDH1	Spo21995	SpoScf_02896	22145	23389	For	1245	414	0
11	SoMDH2	Spo08175	chr5	10849769	10851010	Rev	1242	413	0
12	SoMDH3	Spo10516	chr4	62141844	62145553	For	1032	343	6
13	SoMDH4	Spo22090	SpoScf_03526	16484	21730	Rev	1077	358	7
14	SoCTS1	Spo11084	chr4	101756403	101770489	Rev	1800	599	19
15	SoCTS2	Spo11913	chr4	7539409	7552423	For	1608	535	20
16	SoACO	Spo13736	SpoScf_03007	28088	36725	Rev	2967	988	18
17	SoICL	Spo13898	SpoScf_00215	416756	427351	For	1989	662	6
18	SoAAE3	Spo04424	chr3	3466032	3471417	For	1575	524	3
19	SoOXO1	Spo14475	chr2	40519401	40522642	Rev	666	221	1
20	SoOXO2	Spo04401	chr3	2862241	2863645	For	654	217	1
21	SoOXDE	Spo00223	chr1	31229950	31233690	Rev	1722	573	1
22	SoFXDE	Spo19843	chr4	119024460	119033349	Rev	1911	636	8
23	SoOXDC1	Spo06441	chr4	49505283	49512025	For	1512	503	4
24	SoOXDC2	Spo19759	chr5	17648569	17649072	For	504	167	0
25	SoOXDC3	Spo25084	chr4	4971997	4972967	Rev	441	146	1

## Supplementary Materials

The following are available online. Supplementary Table S1: the genes involved in the oxalate biosynthesis used in this study. Supplementary Table S2: the primers used in this study. Supplementary Table S3: expression data of putative oxalate related genes in spinach. Supplementary Table S4: expression data of putative oxalate related genes under NO_3_^−^ and NH_4_^+^ treatments. Supplementary Figure S1: expression profiles of genes involved in glycolate/glyoxylate oxalate biosynthesis pathways in spinach. The four parts were as follows: (1) mature leaf laminas; (2) mature leaf petioles; (3) young leaf laminas; and (4) young leaf petioles. Error bars represent standard deviation among three real-time quantitative PCR reaction replicates. Data are means of three replicates ±SD. Different lowercase letters indicate significant differences at *P* < 0.05. Supplementary Figure S2: expression profiles of genes involved in glycolate/glyoxylate oxalate biosynthesis pathways under NH_4_^+^ and NO_3_^−^ treatments in leaf laminas of spinach. Error bars represent standard deviation among three real-time quantitative PCR reaction replicates. Data are means of three replicates ±SD. Different lowercase letters indicate significant differences at *P* < 0.05.
