# Supplementary material for: Expression Analysis of Oxalate Metabolic Pathway Genes Reveals Oxalate Regulation Patterns in Spinach
Source: Molecules. 2018 May 27;23(6):1286. doi: 10.3390/molecules23061286 (PMC6100029; doi:10.3390/molecules23061286)
Supplement: Supplementary file 1 [file molecules-23-01286-s001.zip › Supplementary Table S1.docx]

| Supplementary Table S1: the genes involved in the oxalate biosynthesis used in this study | | | |
| --- | --- | --- | --- |
| Number as shown in Fig. 1 | Gene name | | Accesion |
| 1 | Glycolate oxidase | OsGLO | AK098878 |
| 2 | Oxaloacetate acetylhydrolase | BcOXAC | AAS99938 |
| 3 | Malate synthase | AtMLS | AED90664 |
| 4 | Malate dehydrogenase | AtMDH | AEE78292 |
| 5 | Citrate synthase | AtCTS | AAK62463 |
| 6 | Aconitase | CsACO | KGN48958 |
| 7 | Isocitrate lyase | OsICL | AEE76544 |
| 8 | Ascorbate peroxidase/oxidase | AtAPX/CmAO | BAA03334/BAA09528 |
| 9 | Oxalyl-CoA synthetase | AtAAE3 | AF503762 |
| 10 | Oxalate decarboxylase | FvOXDC | AAF13275 |
| 11 | Oxalate oxidase | AhOXO | ABS86851 |
| 12 | Oxalyl-CoA decarboxylase | AtOXDE | CAC19854 |
| 13 | Formyl-CoA hydrolase | AtFXH | AER67546 |
| 14 | Formate dehydrogenase | AtFXDE | AED92076 |
