# Supplementary material for: Expression Analysis of Oxalate Metabolic Pathway Genes Reveals Oxalate Regulation Patterns in Spinach
Source: Molecules. 2018 May 27;23(6):1286. doi: 10.3390/molecules23061286 (PMC6100029; doi:10.3390/molecules23061286)
Supplement: Supplementary file 1 [file molecules-23-01286-s001.zip › Supplementary Table S2.docx]

| Supplementary Table S2: the primers used in this study | | |  |
| --- | --- | --- | --- |
| Gene name | Unigene number | Forward primer Sequence 5'-3' | Reverse primer Sequence 5'-3' |
| SoGLO1 | Spo19861 | TTGAGGGTATTGACCTGGGC | AGCTCAGGGATCGATCAATCT |
| SoGLO2 | Spo21903 | TGAGGGGATGGATCTTGGT | TCCAATTCAATGACCGGTCA |
| SoGLO3 | Spo10076 | AATGATTGCACCTCAGGTCA | TTTGCAAAAGCTTGAGGACC |
| SoGLO4 | Spo21282 | CTTAGTGCACTGCTGGGGT | GAAGCCAGCCCTCTCAGC |
| SoGLO5 | Spo20781 | GCCAGAGCAGCATCAGCA | TCGGATGCCTGGTCCTGT |
| SoOXAC1 | Spo00571 | CGCCGCTGCTAAACCACT | ACATCGTCTTGGTCCCGC |
| SoOXAC2 | Spo21624 | GCTAGCAATGTGGCTGAAGG | ACAAATGGCGGCGGAGAA |
| SoOXAC3 | Spo21589 | GCTGCCGCTCCTCTAGGG | ACAAATGGCGGCGGAGAA |
| SoMLS | Spo16696 | GTGCCTCAAGGGGTGGAC | TGGCTCTTAAACTCCCTTTGT |
| SoMDH1 | Spo21995 | GCATCCTGCAATGTGAAGGT | TGCTGCTGCTTTAAGACCAC |
| SoMDH2 | Spo08175 | TGCATCCACCTATGTCAATCC | CACTGAAACCCCTGAGAGG |
| SoMDH3 | Spo10516 | TCTGTGCAATCAGCCGCT | CGCCGAGAATGGTGACCT |
| SoMDH4 | Spo22090 | ATCGGAGGCGAATCAGCG | TGCCCCTTTTGCTCTGCA |
| SoCTS1 | Spo11084 | GGCGCAAGTAAGTTCTGGG | GGACGAGCAATTTCCCCTGA |
| SoCTS1 | Spo11913 | GCAGGCGCAAGGAACATC | GCATCATCCCAAACAGCCT |
| SoACO | Spo13736 | GGTGGCGGTGAATTCGGA | TGGCCGACTCCAAGAGGA |
| SoICL | Spo13898 | GCGCACCCTTAAGAGCCA | GCCACCCCGACACGTAAA |
| SoAPX1 | Spo03202 | CTGACCCTGTCTTCCGCC | AAAGTTTCAAGTGCGCCTCA |
| SoAPX2 | Spo13500 | CCAAGCGGAGGCAAGTCA | ACAAATGACCACTGCTGCAG |
| SoAPX3 | Spo13008 | GAAGCTCTCAGAGTTGGGGT | GTAAGAGTGACCACAACTCCA |
| SoAPX4 | Spo08328 | GCAGCGACGAGGTTGAGT | GCCTGGGCAGCAACCATT |
| SoAPX5 | Spo07617 | CACACGCACAGTTGAAAGCA | GCAAGATTGGAGCAAACTGC |
| SoAPX6 | Spo23721 | TTCACTACCACCACCGCC | GTGAGGAGGAGGAGGAGGA |
| SoAPX7 | Spo19209 | ACCATCGACTTTGTTCCAGG | CTCTCAAATGTCCGGCACCT |
| SoAO1 | Spo22971 | GCCAAGTCAAGGCCAAGGA | GCCTTGCCTTTGCTTCCG |
| SoAO2 | Spo10268 | CCGGAACTCCAACGGGTC | AGTCGTTCCACATCGGGC |
| SoAAE3 | Spo04424 | TGGGTCAGGAGATGGCGA | TTGTGACATTCGGGCCCC |
| SoOXO1 | Spo14475 | AACAACACCATGGGAGCCC | GTCAATACGGCCCATGGAT |
| SoOXO2 | Spo04401 | TCGCCGGAATTTCACAACC | TAAGGCCCGGGATGTCCA |
| SoOXDE | Spo00223 | GGTGGGAAGTCCTGCACC | GGTTCCCCAAGTACCGGC |
| SoFXDE | Spo19843 | ACTGTTGGTGCAGGACGG | CGGGGTCCATCCTGATACG |
| SoOXDC1 | Spo06441 | TCGTCGCCTCCGGGATAA | GAACCCATGCCCTGCACT |
| SoOXDC2 | Spo19759 | CTTTAGCGGGCCTGTCGT | CCTCCACCATCCACCACC |
| SoOXDC3 | Spo25084 | GGACGCCGTCAAGTCTTCA | TGTGCTTGCTGTACCGGG |
