# Supplementary figures and images for: Expression Analysis of Oxalate Metabolic Pathway Genes Reveals Oxalate Regulation Patterns in Spinach
Source: Molecules. 2018 May 27;23(6):1286. doi: 10.3390/molecules23061286 (PMC6100029; doi:10.3390/molecules23061286)

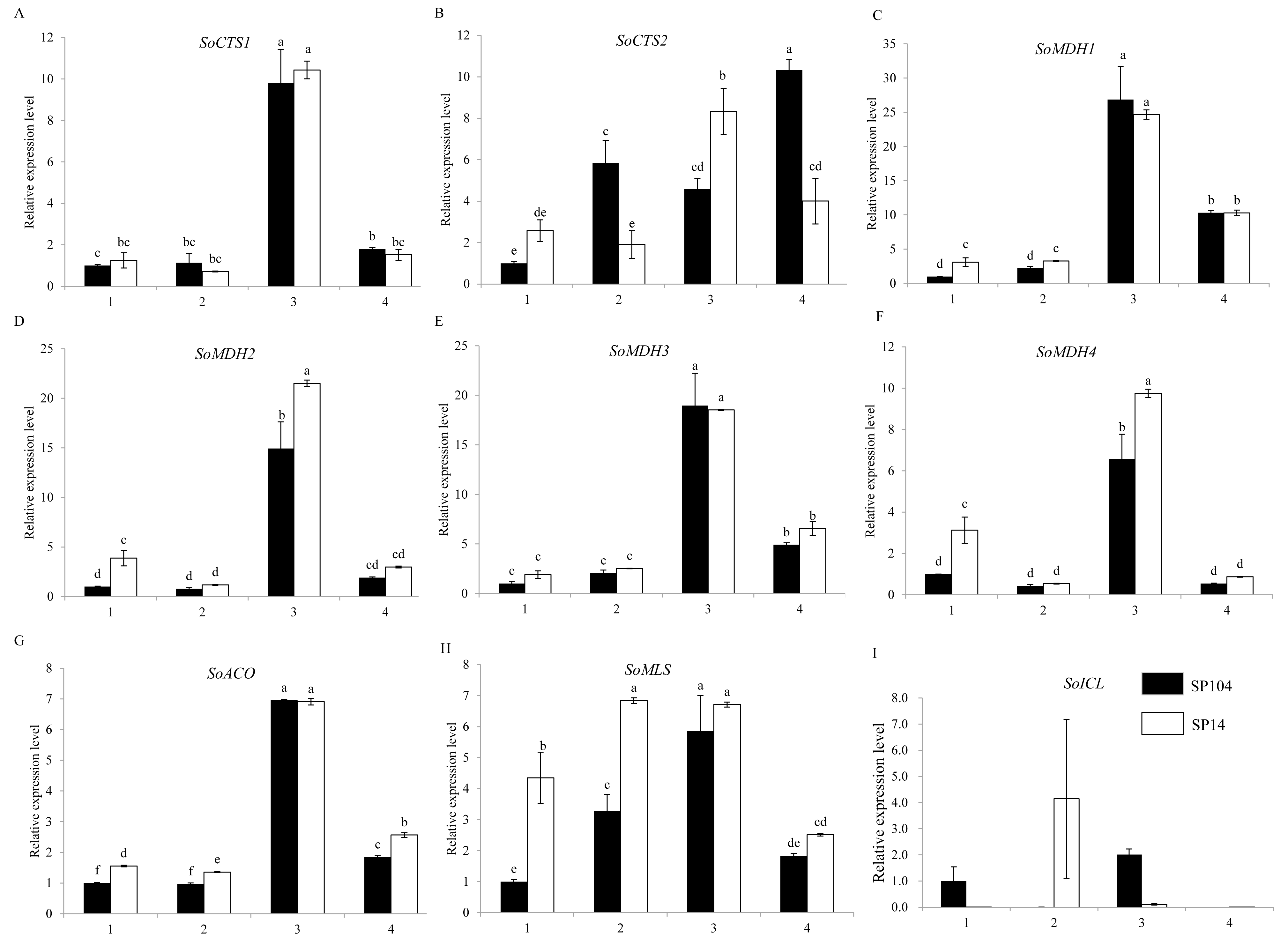

Supplement: Supplementary file 1 [file molecules-23-01286-s001.zip › Supplementary Figure S1.tif]

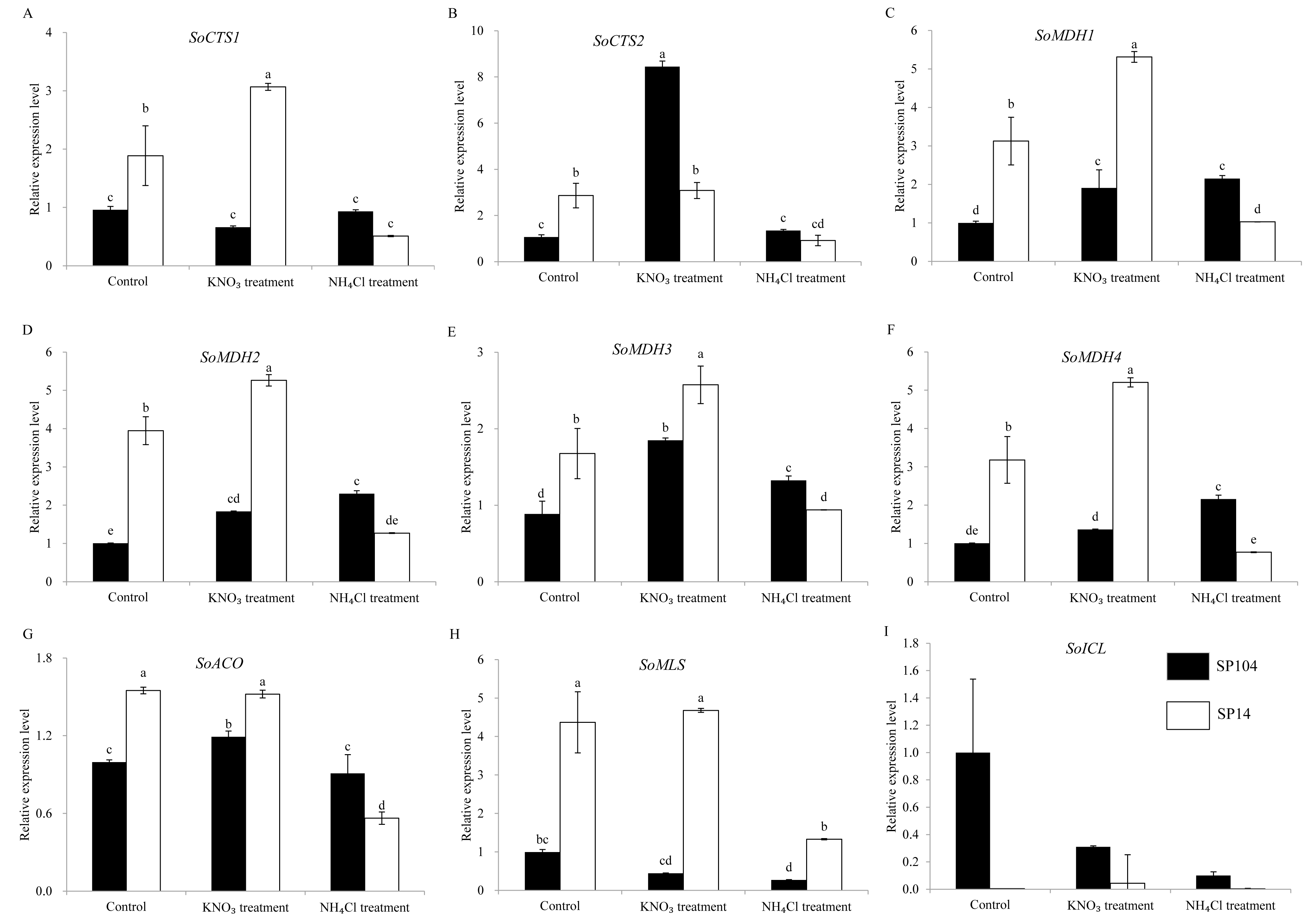

Supplement: Supplementary file 1 [file molecules-23-01286-s001.zip › Supplementary Figure S2.tif]
